# Microstructural damage sensitivity prediction using spatial statistics

**DOI:** 10.1038/s41598-019-39315-x

**Published:** 2019-02-26

**Authors:** B. C. Cameron, C. C. Tasan

**Affiliations:** Department of Materials Science and Engineering, Massachusetts Institute of Technology, Cambridge, USA

## Abstract

The vast compositional space of metallic materials provides ample opportunity to design stronger, more ductile and cheaper alloys. However, the substantial complexity of deformation micro-mechanisms makes simulation-based prediction of microstructural performance exceedingly difficult. In absence of predictive tools, tedious experiments have to be conducted to screen properties. Here, we develop a purely empirical model to forecast microstructural performance in advance, bypassing these challenges. This is achieved by combining *in situ* deformation experiments with a novel methodology that utilizes n-point statistics and principle component analysis to extract key microstructural features. We demonstrate this approach by predicting crack nucleation in a complex dual-phase steel, achieving substantial predictive ability (84.8% of microstructures predicted to crack, actually crack), a substantial improvement upon the alternate simulation-based approaches. This significant accuracy illustrates the utility of this alternate approach and opens the door to a wide range of alloy design tools.

## Introduction

The field of physical metallurgy involves the creation of high strength and high ductility alloys, by relating the spatial characteristics of metal microstructures (e.g. grain size, morphology or crystallography) to their macroscopic properties^1,2^. Both microstructure and property characterization tools have substantially advanced during the last two decades, improving our understanding of this relationship^3–5^. Yet, following a century of research, even with the most powerful physics-based approaches^6,7^, quantitative prediction of the response of complex commercial microstructures^2,8^ remains exceedingly difficult^9,10^. Here, we propose an empirical approach which does not rely on any microstructural or deformation assumptions, giving it wide ranging utility in designing complex commercial alloys. We demonstrate its predictive ability on crack nucleation in a complex duel-phase (DP) steel, an alloy that is used widely in the automotive industry with complex micro-deformation mechanisms^11^.

**Figure 1:**
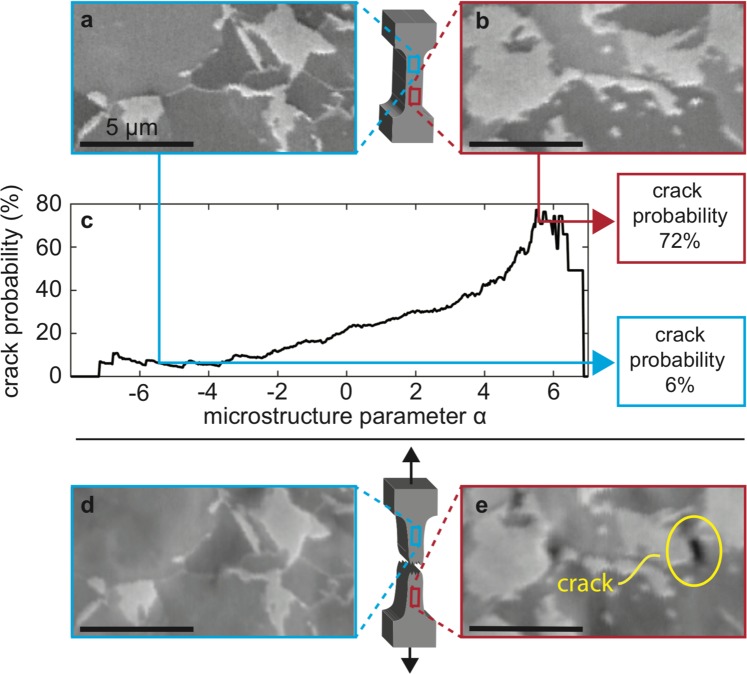
Model making predictions on two microstructures. Cropped images of two microstructural zones before (**a,b**) and after (**d,e**) deformation. Only one cracks (red outline), while the other does not (blue outline). (**c**) The microstructure parameter *α* is extracted from the microstructural geometry and used to predict the probability of cracking upon deformation.

**Figure 2:**
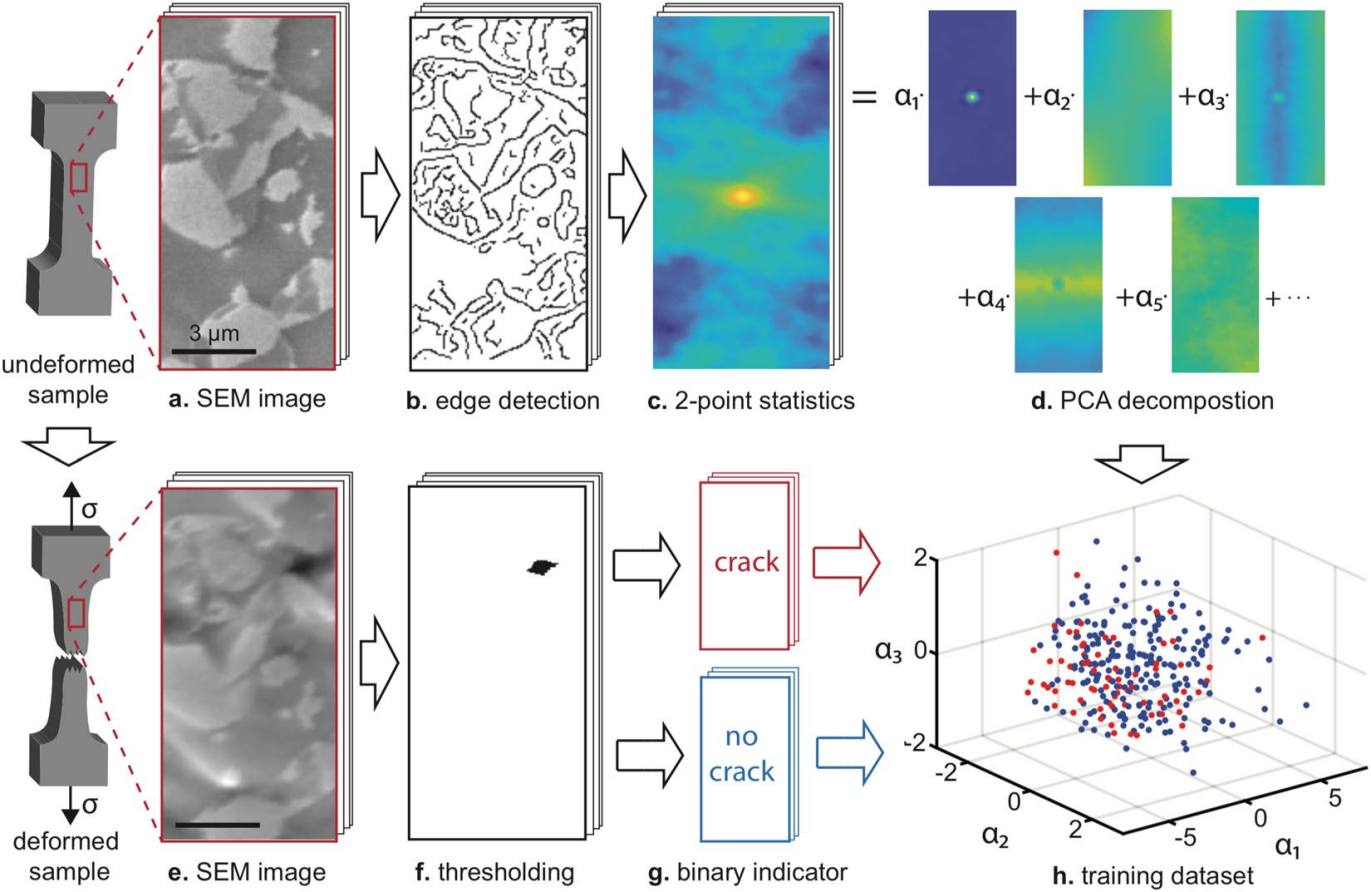
Overview of the developed methodology. Steps to extract the microstructural geometry parameters α_i_ from the undeformed microstructure are shown in (**a–d)**, and the extraction steps of crack data from the deformed microstructure are shown in (**e–g)**. The generated dataset shown in (**h)** can be used to train various statistical models. Note that in (**h)**, each point corresponds to a different microstructure, and that the microstructures shown at (**a**,**e)** are cropped from those actually used.

An example of the crack prediction capability of the model is shown in Fig. 1 for two microstructural zones from a single sample, both containing the martensite (light gray) and ferrite (dark gray) phases typical of dual-phase steels^11^ (Fig. 1a,b). As seen in Fig. 1c, each zone is reduced to a geometry parameter *α*, which is explained in detail below. Strikingly, a clear relationship is seen between *α* and cracking probability. For example, the zone in Fig. 1a that has a low *α* value does not crack (Fig. 1d), and the zone in Fig. 1b that has a high *α* value does crack (Fig. 1e). This first demonstration of cracking susceptibility prediction from microstructure geometries is realized by employing the novel methodology which is schematically described in Fig. 2 and explained in detail next.

To obtain our data, dogbone shaped samples of DP600 steel were metallographically prepared, imaged using electron microscopy, deformed in tension, and re-imaged (for details, see Methods, and Supplementary Fig. 2). In order to ensure a sufficient number of data-points, four large 500 × 500 *μ*m regions were imaged, then subsequently split into 1600 smaller 12.5 × 12.5 *μ*m images during post processing. 960 of these tracked zones are used for training, 320 are used for validation, and 320 are used for testing. Each data-point requires information both before and after deformation, hence, a number of points on the microstructure were tracked so that smaller images corresponding to the same region, both before and after deformation, could be compared (Supplementary Fig. 3). Following this, our dataset is comprised of 1600 data-points, each with an image before deformation, which is used to make a prediction, and an image after deformation, which gives information on what is being predicted (in this case crack nucleation).

The first step in creating a predictive model is to extract key microstructural features of a given microstructural zone (Fig. 2a). Metal microstructures are intrinsically heterogeneous, stochastic and unique^12,13^. In fact, even in a simple single-phase microstructure, thousands of variables would be required to specify the microstructural geometry of even a small region due to the presence of vast range of grain shapes and sizes. Fortunately, several methods have been proposed for extracting key microstructural features, such as lineal path functions^14^, nearest neighbor distributions^15^ and entropic descriptors^16^. We employ the most general and powerful approach, n-point statistics^17–20^, which can be systematically increased in complexity to capture any order of spatial statistics required. However, before this approach is applied, the large number of gray values are reduced to a more physical variable. To this end, we utilize a binary grain boundary variable (rather than the phase variable)^12,17^, obtained from the raw microstructure image by applying Weiner filtering and Canny edge detection consecutively (Fig. 2b)^21^. The sensitivity value is chosen in order to maximize the predictive ability of the algorithm using the validation dataset, giving rise to a surprisingly high number of grain boundaries and noise.

Following edge detection, an n-point statistics transformation^12^ is conducted so that different microstructures with similar geometric features can be easily compared (Fig. 2c). Note that one-point statistics simply gives the probability that a point is on a grain boundary, i.e. the volume fraction of grain boundaries. Two-point statistics gives the probability that two points, a distance ***r*** apart, are both on a grain boundary:

1 $$f({\boldsymbol{r}})=\frac{1}{A}\int I({\boldsymbol{x}})\times I({\boldsymbol{x}}+{\boldsymbol{r}})d{\boldsymbol{x}},$$

where *A* is the total area and *I*(*x*) is the indicator function that is one when there is a grain boundary and zero otherwise. The transformed microstructure *f*(***r***) enables easy extraction of parameters such as the grain size and anisotropy from its length scales, and its two-dimensional space also captures subtler and complex geometric structures than individual parameters such as grain size (Fig. 2c). Only two-point statistics, not three-point or higher order statistics, are considered as this is sufficient to entirely specify the microstructure^17^.

There are still several thousand variables associated with each transformed microstructure. However, there are strong correlations between the pixels which can be exploited using principal component analysis (PCA)^22–24^. Each transformed microstructure *f*_*i*_ can be represented as a linear combination of basis images *g*_*j*_, ${f}_{i}=\sum _{j}\,{\alpha }_{i,j}{g}_{j}$ for $1\le i\le 960$ and $1\le j\le 960$ (Fig. 2d). The variance *λ*_*j*=*J*_ rapidly decays with increasing *J* (e.g. *λ*_2_/*λ*_1_ = 0.19, *λ*_10_/*λ*_1_ = 0.0037 and *λ*_100_/*λ*_1_ = 0.00022); hence, only the first few *α* parameters are required to describe the vast majority of the variance between microstructures. After PCA, each microstructure can be simply represented as a data point with associated *α*_1_, *α*_2_ and *α*_3_, or any other number of *α*_*j*_ values (where the *i* index has been removed) (Fig. 2h). To compute the *α*_*j*_ value for a microstructure, the microstructure is preprocessed, subjected to edge detection, transformed via two-point statistics, and it is projected onto the *g*_*j*_ basis image. Note that when implementing this algorithm only the training data is used to compute *g*_*j*_, as the test data is reserved to assess the predictive ability.

A binary variable indicating the existence of a crack is obtained by simply checking how many pixels are darker than a certain gray value (Fig. 2e–g). This gray value is chosen to correspond to the existence of physical cracks, based on extensive SEM imaging and analysis discussed in prior papers^25–27^. The predictive ability of the algorithm is not sensitive to the specific value. Note also that in this work our focus is on micro-crack prediction, yet there is no reason why other parameters such as damage size, growth susceptibility, and strain localization events could not also be successfully predicted following the same methodology (Fig. 2).

Each microstructural region is now characterized by a set of *α*_*j*_ values that are obtained from the microstructure before deformation and a binary variable *y* indicating the existence of a crack after deformation (Fig. 2h). This opens the door to numerous statistical and/or machine learning models to relate these parameters, such as support vector machines, neural networks and logistic regression models. Here, we simply consider the *α*_1_ value as a proof-of-principle demonstration of the approach (referred to as the *α* value in the remainder of the text). Probability distribution functions *P*(*α*|*y*) are computed as a function of *α* for all microstructures with cracks *P*(*a*|*y* = 1) and without cracks *P*(*a*|*y* = 0)using kernel density estimation. This can then be used to calculate the probability that a new microstructural region will have a crack from its *α* value:

2 $$P(y|\alpha )=\frac{P(\alpha |y)P(y)}{P(\alpha )}.$$

This equation is used to calculate the curve shown in Fig. 1c, which can be used to make predictions about the behavior of new microstructures. When the model is tested on new microstructures, 5% of microstructures that are assigned crack probabilities less than 10% cracked, and 38% of microstructures that are assigned crack probabilities greater than 40% cracked, indicating slight overfitting. Here, we report on the 40% value as this is the highest value that could be chosen while retaining a sufficient amount of data to compute statistics. See Supplementary Table 1 and Supplementary Fig. 4 for more extended results on the performance of the model.

**Figure 3:**
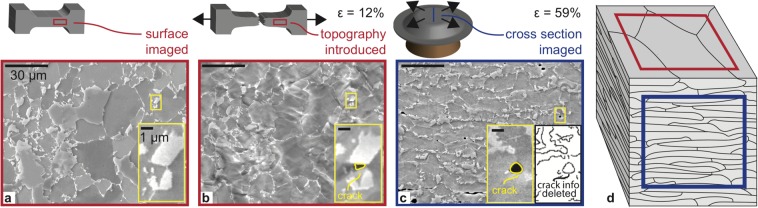
*In-situ* and *post-mortem* deformation data. (**a**,**b**) Original experiment where the sample surface is imaged before and after deformation, respectively. (**c**) Modified experiment where the sample is deformed and a cross section is imaged in a region of high strain. (**d**) Imaging the surface (red square) and cross section (blue square) result in different amounts of information about the internal microstructure.

While the employment of the described framework led to substantial predictive ability, there is still room for improvement. For this purpose, it should be considered that (i) most damage incidents in DP steels are observed within the localized neck, where the local strain can exceeds 50%^11^; and (ii) in absence of free surface relaxation effects, stress build-up leads to damage nucleation at lower strain values for the bulk^28^. We next demonstrate that two changes in the experimental methodology, namely, testing to higher strains and focusing on bulk damage, are sufficient to increase crack prediction probability by leading to larger and more numerous crack generation. However, *in situ* SEM testing does not allow these changes, since the plastic deformation leads to significant surface roughening, impairing the ability to image cracks (Fig. 3a,b). Therefore, we measure damage incidents *post mortem* over a sample cross section after deforming it to higher strains. Specifically, the DP steel is subjected to a flat punch test^29,30^ and the imaging is conducted at a location with a local von Mises strain of 59.9%, a significant increase from the 12% strain achieved during the *in situ* test (Fig. 3c). We solve the main limitation of this approach, i.e. the absence of the undeformed state image, by using the deformed microstructure image and relying on the fact that edge detection deletes crack information, rendering it indistinguishable from a grain boundary (Fig. 3c inset and Supplementary Note). This ensures that the crack information is not used to predict cracking. This approach leads to a four-fold increase of crack density (1.91 × 10^−3^ to 8.76 × 10^−3^ cracks/μm^2^) as aimed, giving more data points upon which to train the model. Also note that imaging the cross section of this anisotropic material gives more information about its internal structure (Fig. 3d), as can be seen by comparing Fig. 3a,c. The increased information may result in improved predictive ability, for example, there is a lower chance that hidden sub-surface martensite grains will result in unexpected crack nucleation events.

**Figure 4:**
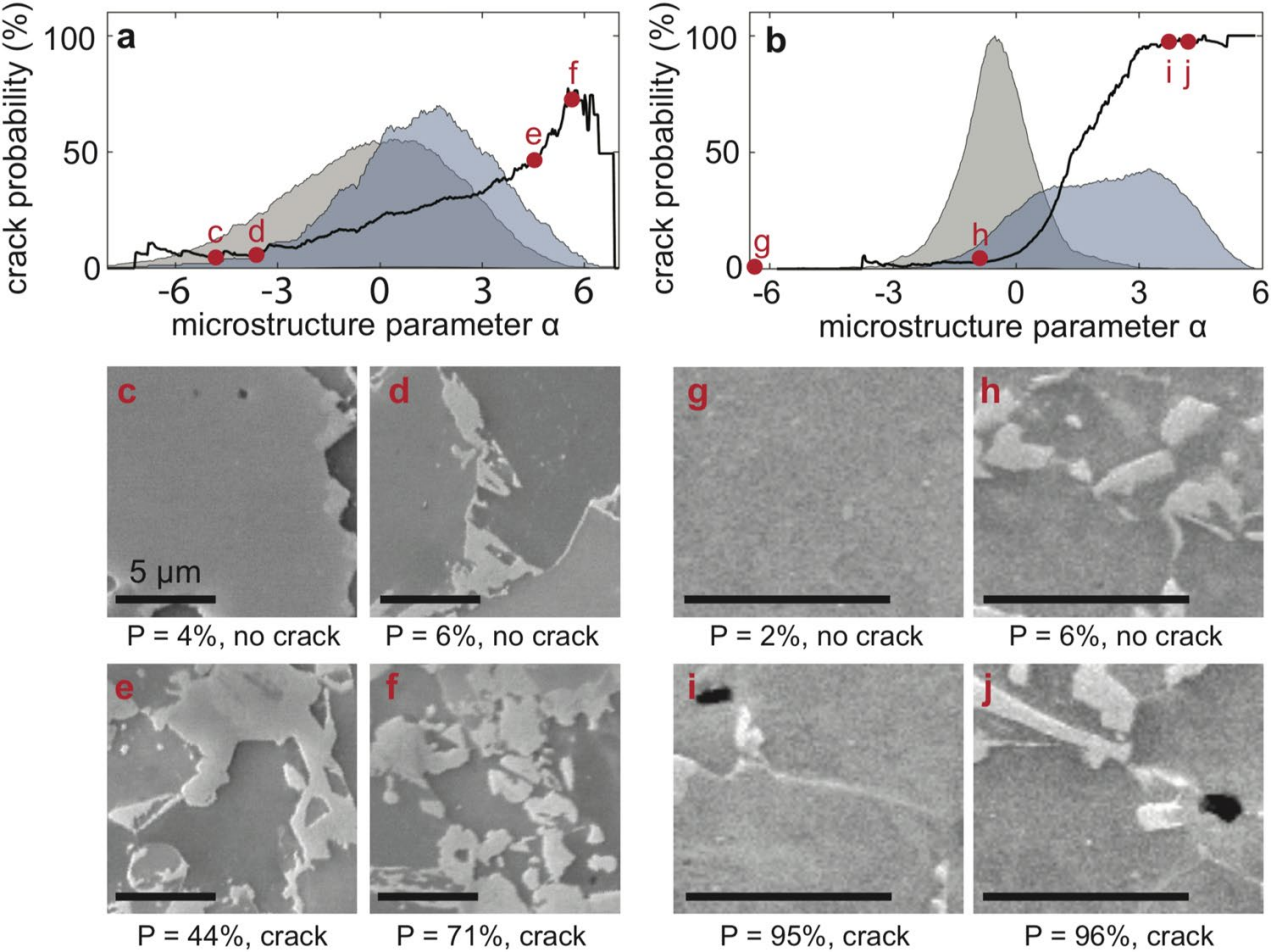
Overview of the results obtained in this study. **(a)** Using the dogbone sample dataset and (**b)** flat punch dataset; and corresponding microstructure examples in (**c–j)**, respectively. In (**a**,**b)**, the probability density of data with a crack (blue) and without a crack (gray) can be seen. The difference in these distributions can be exploited to calculate the probability of cracking as a function of α (black).

These changes in the methodology result in significantly improved predictive ability (Fig. 4a,b). The probability distributions for data with and without a crack are further separated from one another and the crack predictions are much closer to 100% or 0% for a larger range of *α* values. When the model is tested on new data, we find that of the microstructures assigned crack probabilities greater than 90% by the model, 99.2% were observed to crack; of the microstructures that were assigned crack probabilities less than 10%, only 4.6% crack. See Supplementary Fig. 5 and Table 2 for more extended results on the performance of the model. The model performs well on both simple and complex geometries, and picks up some non-obvious features that are likely to cause grain boundary cracking (Fig. 4c–j). For example, Fig. 4d,e are predicted to have vastly different damage nucleation probabilities despite both having complex dual-phase structures. Additionally, Fig. 4i has a very high cracking probability despite the low martensitic volume fraction.

In summary, we developed a novel approach based on microstructure statistics, and demonstrated its capabilities by quantitatively predicting cracking in a multi-phase steel. The model is not material specific and can be applied, in its current state, to a broad range of polycrystalline metals and composite materials. It is also highly efficient since the required computational times (e.g. order of an hour, for the present dataset) are vastly lower than typical physically-based multi-scale simulation approaches. Furthermore, the demonstrated predictive ability indicates that prediction of many other micro-events (e.g. micro-plasticity, strain localization) are within reach. A wide range of follow-up studies are thus called for, focusing not only on real but also on artificially-constructed microstructures; and using not only classical imaging methods but more sophisticated micro-mapping techniques (e.g. electron backscatter diffraction and digital image correlation). In this regard, this approach opens the door to a range of new tools and approaches for analyzing and understanding metallic materials.

## Methods

A noncommercial 1 mm thick cold rolled DP600 steel, with chemical composition described in Peters (2011)^29^, was used in collecting both the *in-situ* and *post-mortem* datasets. The *in-situ* dataset was obtained using the following methodology. Dogbone shaped tensile samples were waterjet cut from the steel sheet metal. The sample was ground, polished using an OPS suspension and etched using the same methodology^30^. Prior to deformation, four 500 × 500 um images were imaged using a Tescan Mira 3 electron microscope with a working distance of 15 mm, a beam current of 10 nA, an accelerating voltage of 30 kV and a SE detector. A depth mode and large working distance of 15.5 mm was chosen to minimize stigmation at the boundaries of the image. The sample was then deformed using a Gatan stage model MTTEST2000 (Supplementary Fig. 1), at a strain rate 4.4 × 10^−5^ *s*^−1^. Numerous images were made throughout deformation, though only the images before and after deformation were used in data analysis. Finally, after rupture, the same regions originally imaged were re-imaged with a slightly larger view field (square images 560–580 × 560–580 *μ*m) to accommodate the change in shape of the region. Images were collected with an in-lens SE detector and a standard SE detector, though only the standard images were used for crack recognition. The positions of 5–7 points were manually tracked in each imaged region and a linear map using a strain/rotation tensor and a displacement vector was computed using linear regression (Supplementary Fig. 2). The values for this strain/rotation tensor and displacement vector where well converged and did not change substantially when additional points were added. There was a small error of 0–1 *μ*m due to the spatially varying nature of the strain field. This error could be reduced in future work by computing a non-linear displacement map or fully implementing DIC, however because the error was small in comparison to the size of our images this was not conducted. For the analysis presented in the paper, only one image was used as this gave slightly improved results (there were minor differences in the brightness, contrast, and other imaging conditions between images). Each large, macroscopic image was split into 1600 12.5 × 12.5 images, all used as individual data points. The *post mortem* dataset was obtained from Peters (2011)^30^.

The algorithm was implemented in MATLAB, using their implementations of autocorrelation to compute the n-point statistics. The principle component analysis was implemented using the method of snapshots, and only the training dataset is used to compute the basis images^12^. The sensitivity value for the canny edge detection is defined as the ratio of the low gradient threshold and the high gradient threshold, and is optimized at 0.01 increments between 0 and 0.3.

## Supplementary information

Supplementary Material

## Data Availability

The datasets generated during and/or analysed during the current study are available from the corresponding author on reasonable request.
