# Supplementary material for: Microstructural damage sensitivity prediction using spatial statistics
Source: Sci Rep. 2019 Feb 26;9:2774. doi: 10.1038/s41598-019-39315-x (PMC6391476; doi:10.1038/s41598-019-39315-x)
Supplement: Supplementary file 1 — Supplementary Material [file 41598_2019_39315_MOESM1_ESM.docx]

**Supplementary Material**

**Microstructural damage sensitivity prediction using spatial statistics**

**B. C. Cameron and C. C. Tasan***

^1^Department of Materials Science and Engineering, Massachusetts Institute of Technology, USA

*corresponding author: [tasan@mit.edu](mailto:tasan@mit.edu)

**Supplementary note: Edge detection deleting crack information**

This supplementary note addresses the concern that the algorithm is simply recognizing the crack, rather than predicting it from the microstructural geometry as intended. This concern only applies to the *post-mortem* dataset, because when the algorithm is applied to the *in-situ* dataset, only the microstructures from before deformation are used to make the prediction, and significant predictive ability is achieved.

To address this, we make the argument that after edge detection, a crack is indistinguishable from any other grain boundary feature (Fig. 3c inset). Nevertheless, the critical reader may have valid concerns that the crack information is preserved through the edge detection algorithm, and that the improvement in predictive ability while using the *post-mortem* dataset can be attributed to crack recognition. To substantiate our argument, consider Supplementary Figure 1. When comparing the edge detection images of Figure S1a and b, one can see that the crack in Figure S1a appears the same as the grains in b. The same is true for Figure S1c and d.

There are multiple factors that can be used to explain the improvement between the *in-situ* and *post-mortem* datasets, such as anisotropic effect, increased deformation levels and larger numbers of data points discussed in the main article. Nevertheless, we cannot prove that hidden information about the existence of the crack is not a component of this. Further investigations would be required to determine what the various contributions of these factors are. However, the fact that the algorithm still has some predictive ability on the *in-situ* datasets alleviates many concerns.


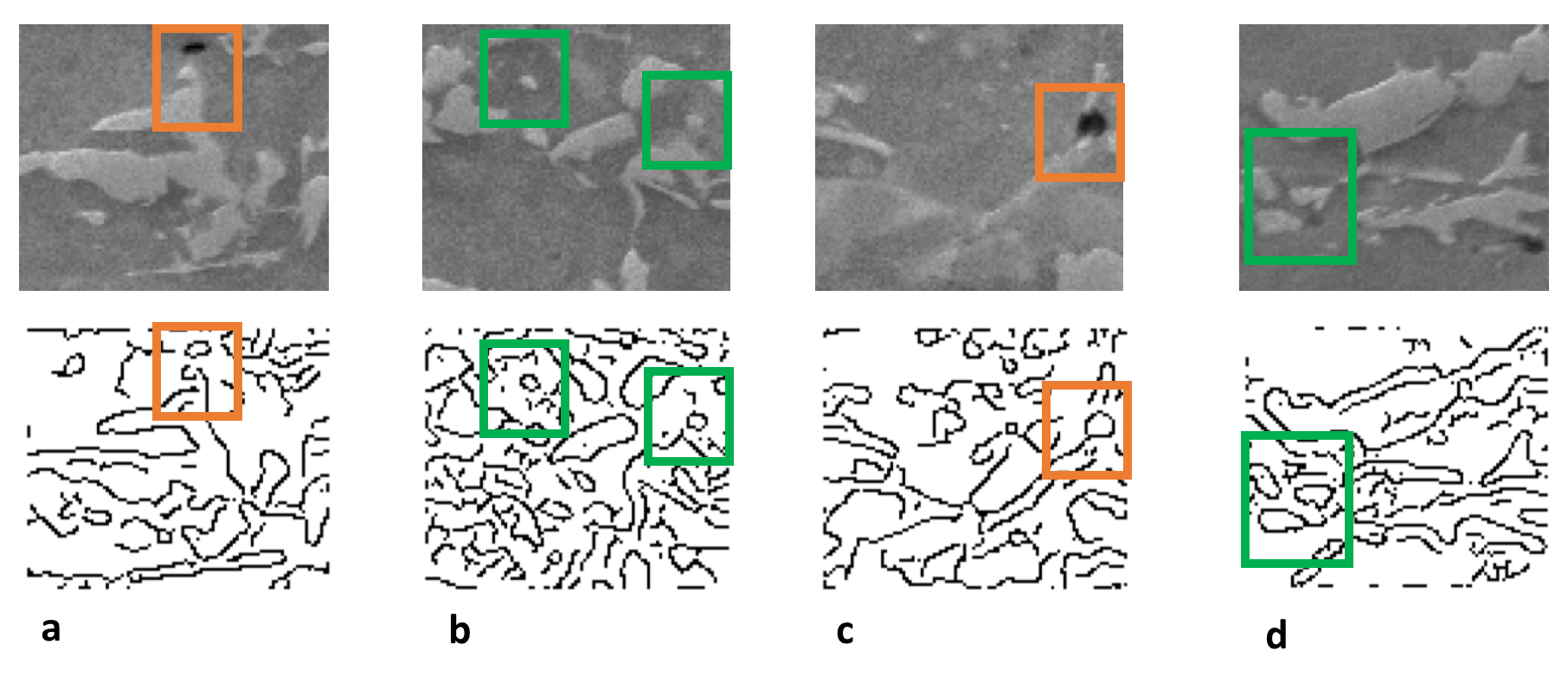


**Figure S1| Edge detection deleting crack information.** Shows examples of microstructures from the *post-mortem* dataset with edge detection applied to the microstructures. The orange boxes highlight the cracks and the corresponding data preserved in the processed image (**a,c**). The green boxes highlight grains that when transformed, appear the same as cracks (**b,d**).


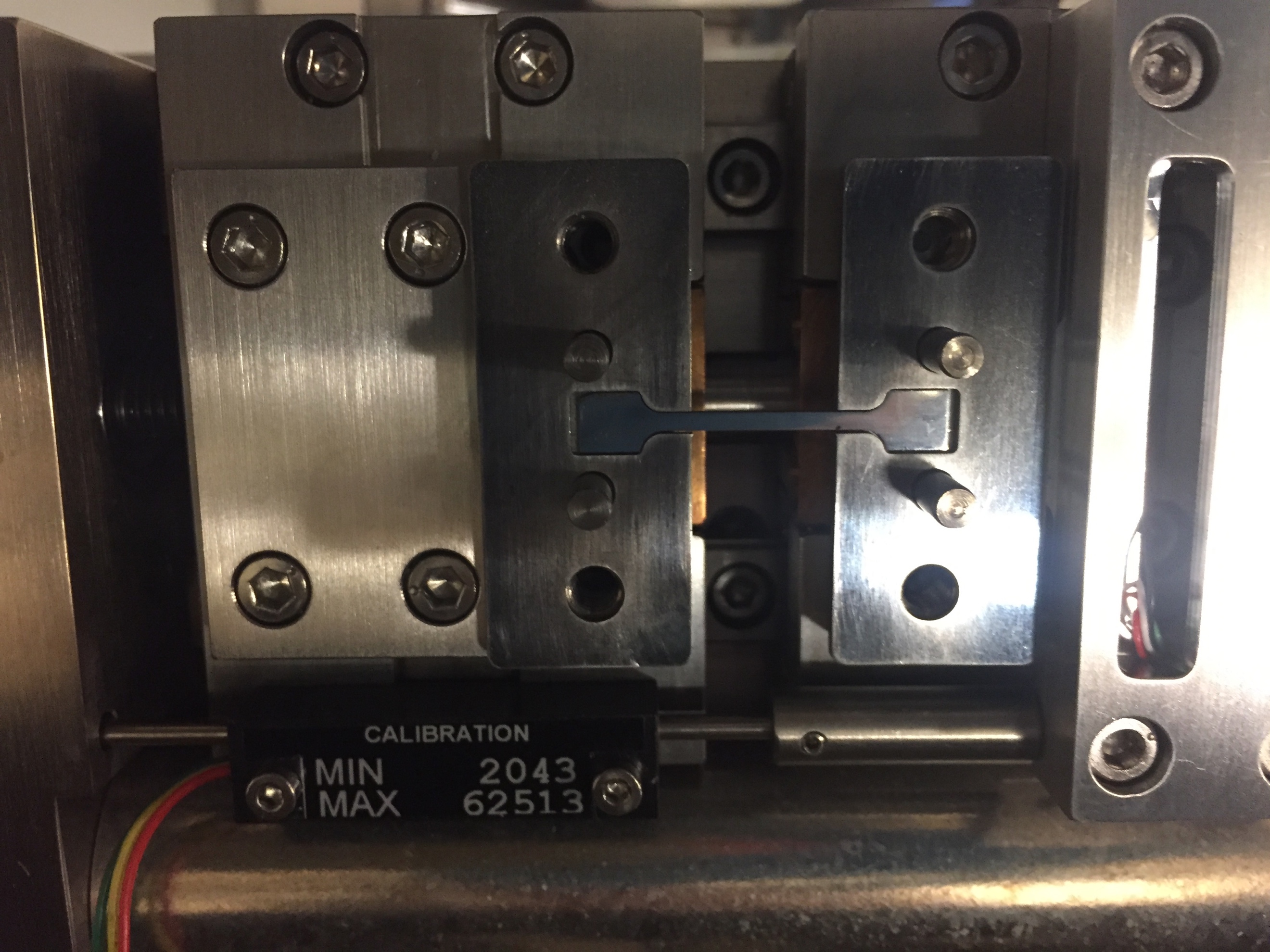


**Figure S2| Sample loaded in *in-situ* deformation stage.** Here the polished sample is partially loaded into a Gatan tensile stage model MTTEST2000. Before beginning deformation the sample is also clamped from above and imaged.


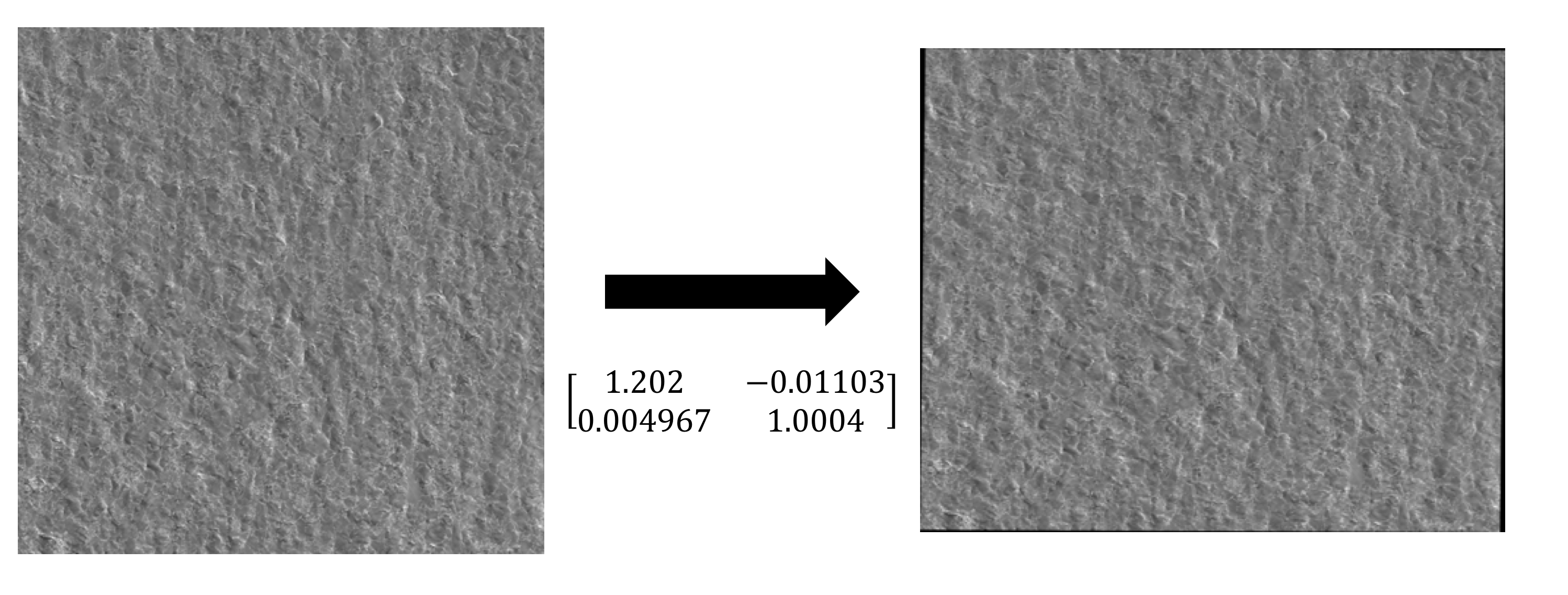


**Figure S3| Transformation of deformed microstructure to original reference frame.** Here, a transformation matrix is computed by tracking approximately 6 points throughout deformation and using linear regression. The matrix is applied to the deformed image to revert it back to the original reference frame so it can be compared with the unreformed image.


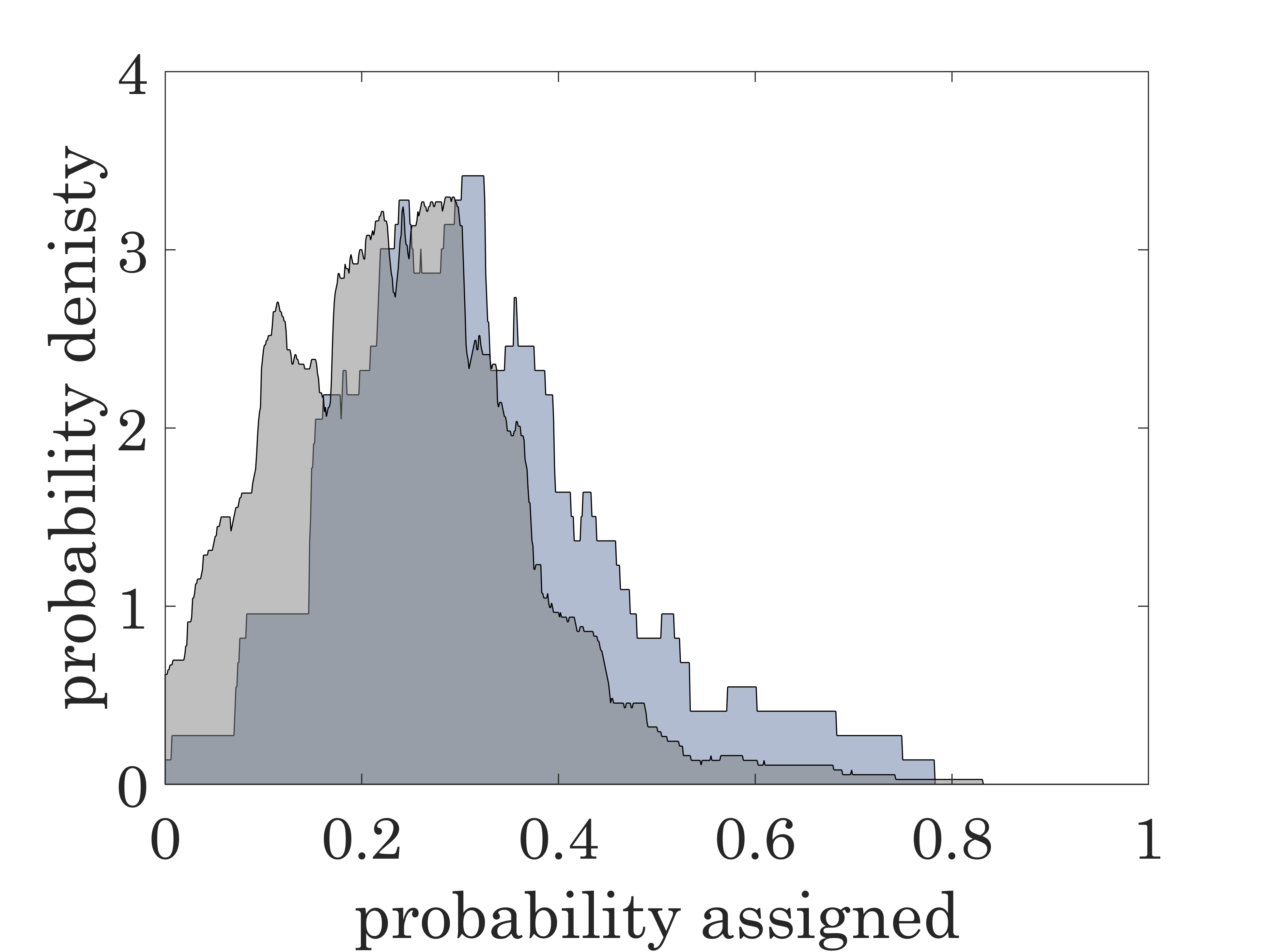


**Figure S4| Distribution of model predictions on *in-situ* data using test microstructures.** The blue and gray probability density functions correspond to test data that cracks and doesn’t crack respectively. The x-axis shows the probability assigned by the model. The distributions are computed using kernel density estimation.


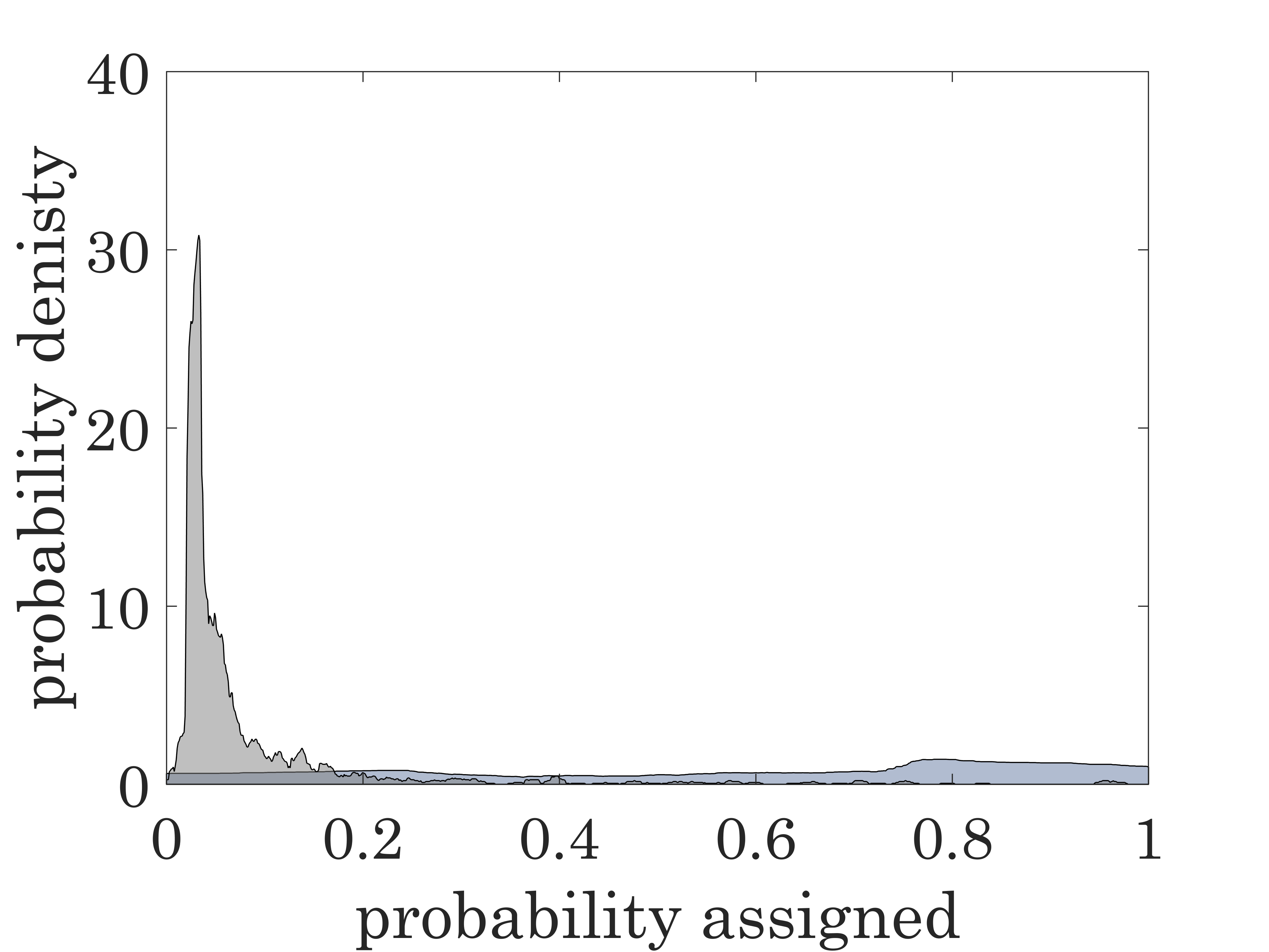


**Figure S5| Distribution of model predictions on *post-mortem* data using test microstructures.** The blue and gray probability density functions correspond to test data that cracks and doesn’t crack respectively. The x-axis shows the probability assigned by the model. This shows substantial improvement over the *in-situ* data. The distributions are computed using kernel density estimation.

**Table S1| Values of model predictions on *in-situ* data using test microstructures.** *some of the microstructures are assigned 0 probability of cracking

| **Probability P (%)** | **Fraction of microstructures assigned probabilities p>P that crack (%)** | **Fraction of microstructures that crack that are assigned probabilities p>P (%)** | **Fraction of microstructures that don't crack that are assigned probabilities p>P (%)** |
| --- | --- | --- | --- |
| 0 | 11.1 | 100.0 | 98.2* |
| 1 | 11.1 | 100.0 | 98.2 |
| 2 | 11.1 | 100.0 | 98.2 |
| 5 | 11.2 | 100.0 | 97.5 |
| 10 | 13.3 | 97.1 | 77.9 |
| 20 | 17.9 | 82.9 | 46.7 |
| 30 | 25.3 | 54.3 | 19.6 |
| 40 | 58.3 | 20.0 | 1.8 |
| 50 | 50.0 | 8.6 | 1.1 |
| 60 | 100.0 | 2.9 | 0.0 |
| 70 | 100.0 | 2.9 | 0.0 |
| 80 | - | 0.0 | 0.0 |

**Table S2| Values of model predictions on *post-mortem* data using test microstructures.** *some of the microstructures are assigned 0 probability of cracking

| **Probability P (%)** | **Fraction of microstructures assigned probabilities p>P that crack (%)** | **Fraction of microstructures that crack that are assigned probabilities p>P (%)** | **Fraction of microstructures that don't crack that are assigned probabilities p>P (%)** |
| --- | --- | --- | --- |
| 0 | 19.1 | 100.0 | 99.5* |
| 1 | 19.2 | 100.0 | 99.3 |
| 2 | 19.7 | 100.0 | 96.0 |
| 5 | 35.1 | 93.8 | 40.8 |
| 10 | 51.8 | 84.6 | 18.5 |
| 20 | 69.2 | 75.1 | 7.9 |
| 30 | 76.5 | 71.5 | 5.2 |
| 40 | 82.4 | 67.5 | 3.4 |
| 50 | 84.8 | 64.3 | 2.7 |
| 60 | 88.5 | 58.0 | 1.8 |
| 70 | 90.4 | 52.8 | 1.3 |
| 80 | 94.7 | 41.3 | 0.5 |
| 90 | 94.8 | 35.7 | 0.5 |
| 95 | 94.1 | 31.1 | 0.5 |
| 98 | 100.0 | 5.9 | 0.0 |
| 99 | 100.0 | 1.0 | 0.0 |
